# Capturing how age-friendly communities foster positive health, social participation and health equity: a study protocol of key components and processes that promote population health in aging Canadians

**DOI:** 10.1186/s12889-017-4392-7

**Published:** 2017-05-25

**Authors:** Mélanie Levasseur, Marie-France Dubois, Mélissa Généreux, Verena Menec, Parminder Raina, Mathieu Roy, Catherine Gabaude, Yves Couturier, Catherine St-Pierre

**Affiliations:** 1 School of Rehabilitation, Faculty of Medicine and Health Sciences, Université de Sherbrooke, 3001, 12e Avenue Nord, Sherbrooke, Quebec, J1H 5N4 Canada; 2 Department of Community Health Sciences, Faculty of Medicine and Health Sciences, Université de Sherbrooke, 3001, 12e Avenue Nord, Sherbrooke, Quebec, J1H 5N4 Canada; 3 Research Centre on Aging, Centre integré universitaire de santé et de services sociaux de l’Estrie — Centre hospitalier universitaire de Sherbrooke (CIUSSS de l’Estrie – CHUS), 1036 Belvedere South, Sherbrooke, Quebec, J1H 4C4 Canada; 4 Department of Community Health Sciences, University of Manitoba, S113 Medical Services Building, 750 Bannatyne Ave, Winnipeg, MB R3E 0W3 Canada; 5 McMaster University, 1280 Main Street West, Hamilton, ON L8S 4L8 Canada; 6 CIUSSS de l’Estrie – CHUS, 375, rue Argyll, Sherbrooke, Quebec, J1J 3H5 Canada; 7 Department of Family Medicine and Emergency Medicine, Faculty of Medicine and Health Sciences, Université de Sherbrooke, 3001, 12e Avenue Nord, Sherbrooke, Quebec, J1H 5N4 Canada; 8 Institut français des sciences et technologies des transports, de l’aménagement et des réseaux, 14-20 bd Newton - Cité Descartes, Champs-sur-Marne, 77447 Marne-la-Vallée Cedex 2, France; 9 Department of Social Services, Faculty of Letters and Humanities, Université de Sherbrooke, 2500, boul. de l’Université, Sherbrooke, Quebec, J1K 2R1 Canada

**Keywords:** Aging adults, Seniors, Age-friendly cities, Age-friendly municipalities, Community integration, Community participation, Social engagement, Social involvement, Mixed-method design, Canadian longitudinal Study on Aging (CLSA)

## Abstract

**Background:**

To address the challenges of the global aging population, the World Health Organization promoted age-friendly communities as a way to foster the development of active aging community initiatives. Accordingly, key components (i.e., policies, services and structures related to the communities’ physical and social environments) should be designed to be age-friendly and help all aging adults to live safely, enjoy good health and stay involved in their communities. Although age-friendly communities are believed to be a promising way to help aging Canadians lead healthy and active lives, little is known about which key components best foster positive health, social participation and health equity, and their underlying mechanisms.

This study aims to better understand which and how key components of age-friendly communities best foster positive health, social participation and health equity in aging Canadians. Specifically, the research objectives are to:

1. Describe and compare age-friendly key components of communities across Canada
2. Identify key components best associated with positive health, social participation and health equity of aging adults
3. Explore how these key components foster positive health, social participation and health equity

**Methods:**

A mixed-method sequential explanatory design will be used. The quantitative part will involve a survey of Canadian communities and secondary analysis of cross-sectional data from the Canadian Longitudinal Study on Aging (CLSA). The survey will include an age-friendly questionnaire targeting key components in seven domains: physical environment, housing options, social environment, opportunities for participation, community supports and healthcare services, transportation options, communication and information. The CLSA is a large, national prospective study representative of the Canadian aging population designed to examine health transitions and trajectories of adults as they age. In the qualitative part, a multiple case study will be conducted in five Canadian communities performing best on positive health, social participation and health equity.

**Discussion:**

Building on new and existing collaborations and generating evidence from real-world interventions, the results of this project will help communities to promote age-friendly policies, services and structures which foster positive health, social participation and health equity at a population level.

## Background

### What research on population health interventions should be done?

#### Aging: One of the most important challenges that require innovative population interventions to promote health and health equity of Canadians

In 2014, older adults made up 15.7% of the Canadian population [1] and this proportion is expected to double over the next 25 years. Chronic diseases, such as arthritis and rheumatism (47.3%), hypertension (42.8%), heart disease (19.8%) or diabetes (13.5%), affect many people aged 65 and older and almost half (42%) of them live with disabilities [2]. These demographic challenges are one of the most important factors influencing our society. Fortunately, chronic diseases and disabilities can be prevented or delayed by advanced initiatives, including population health interventions. However, such interventions require means in a context where human resources are limited and there are major financial restrictions [3]. Consequently, the health and quality of life among aging adults is a major concern for decision-makers, professionals and researchers, and innovative and cost-effective population health interventions tackling modifiable determinants of health are needed [4].

#### Positive health, social participation and health equity: Important issues for aging adults

*Positive health* focuses on why some people are healthy and others in the same situation are not [5], i.e., what creates health and strengthens the effectiveness of clinical and population interventions [6]. Measures of positive health include outcomes such as life satisfaction, functional status and performance. Such a salutogenic focus operationalizes the content, values, and principles of the Ottawa Charter for Health Promotion [7]. Health promotion is the process of enabling people to increase control over and improve their health, and focuses on promoting self-esteem and coping abilities of individuals and communities, ultimately leading to less dependence on professional services [8]. Among positive health strategies and inspired by health promotion, the World Health Organization (WHO) Active Ageing Policy Framework defines active ageing as ‘*the process of optimizing opportunities for health, participation and security in order to enhance quality of life as people age*’ [9]. Adopted by the WHO in the late 1990s, this framework recognizes the factors that affect how individuals and populations age [10], and the right to equal opportunities and treatment in all aspects of life as people grow older, including participation in the political process and community life [9]. Health, the labour market, employment, education, urban design and social policies supporting active ageing can potentially result in fewer disabilities associated with chronic diseases or premature deaths, more people participating actively in community life and enjoying a positive quality of life, and lower costs for medical treatment and care services. Active ageing policies and programs encourage personal responsibility, age-friendly environments and intergenerational solidarity [9].

As one of the key dimensions of health and active aging, *social participation* has been found to be a determinant of many health and quality of life outcomes such as mortality [11], morbidity [12], hospitalisation [13], and functional autonomy [14]. While greater social participation has been demonstrated to be positively associated with these outcomes, social isolation, at the other end of the continuum, has been shown to have detrimental health consequences. For example, isolated older adults were found to be at greater risk of being rehospitalised, even when controlling for health status [11]. Social participation can be defined as a person’s involvement in activities that provide interactions with others in the community [15] and results from the interaction between personal and environmental factors [16]. Known to protect against cognitive decline among community-dwelling older persons [17], participating in society is primarily for the person’s own sake and cannot be accomplished by someone else without losing its benefits (e.g., pleasure from being with others) [18]. From a population perspective, older helpers and volunteers are a resource for their families, communities and economies in supportive and enabling living environments [19]. Social participation has been shown to be closely related to mobility in the community [20] and home [21], and to decline as a result of the ‘normal’ aging process [22, 23]. Facilitated when the abilities of the person and the environment are optimised [24], social participation can be enhanced by population [25] and individual [26] interventions.

*Health equity* is the absence of unfair systems and policies that cause health inequalities, i.e., presence of groups of people that are at greater risk of experiencing poorer overall health than the general population [27]. Health outcomes are stratified by social contexts [28], which engender differential exposure and vulnerability to health-damaging conditions, and disparity in economic and social consequences [29]. Health inequalities emerge from the accumulation of exposures in different degrees and from everyday life situations that generate threat, fright and coping difficulties [29]. The main groups of factors that have been identified as playing an important part in explaining health inequalities are *material* factors (economic and physical environments, including housing conditions) [30], *psychosocial* stressors (e.g., negative events, stressful circumstances, lack of support), and *behavioural* factors (passive or active smoking [31], diet, alcohol consumption, physical exercise, etc.) [29]. Tackling health inequalities involves addressing the unequal distribution of these health determinants [32], such as income and social status, education and literacy, physical environments, social supports and coping skills, healthy behaviours, access to health services [27]. Health equity seeks to reduce inequalities by increasing access to opportunities and conditions conducive to health for all by 1) improving living and working conditions, 2) tackling the inequitable distribution of power, money and resources, 3) measuring and understanding equity and assessing the impact of action, and 4) enhancing health promotion and disease prevention policies. Health inequalities might be reduced by, for example, reinforcing factors that might lessen susceptibility to health effects from inequitable exposures using various means including empowerment, social support and community development, or strengthening policies that reproduce contextual factors such as social capital that might modify the health effects of poverty [28]. Although policies and interventions have been developed to achieve these goals, a better understanding of how they promote health and well-being in all aging adults is needed.

#### Age-friendly communities: A promising population intervention to enhance positive health, social participation and health equity in aging

In an effort to shape active aging as a lifelong process and take advantage of the potential that older people represent for humanity, the WHO challenged worldwide communities to become more age-friendly [19]. In Canada, all provinces have initiated age-friendly community processes [33] and approximately 800 communities have launched age-friendly initiatives. An age-friendly community encourages active aging by optimizing opportunities for health, participation and security by adapting its structures and services to be accessible to and inclusive of older people with varying needs and capacities [19]. Eight issues and concerns have been voiced by older people as characteristics of an age-friendly community: *1) outdoor spaces and buildings, 2) transportation, 3) housing, 4) opportunities for social participation, 5) respect and social inclusion, 6) civic participation and employment, 7) communication and information, and 8) community support and health services*.

Taking these health-promoting issues into consideration, and in accordance with the theoretical perspective of Glass and Balfour [34], neighbourhood *facilitators* (i.e., helpful environmental factors) can enable greater positive health, social participation and health equity [34, 35]. For example, neighbourhood characteristics such as living close to services [36, 37] like grocery stores, health services, public transportation, banking services and social clubs, have been shown to be important in performing activities to meet daily needs. A neighbourhood perceived as friendly and supportive has also been reported to be independently associated with an increased likelihood of participating in social activities [38]. In contrast, personal capabilities might be challenged or exceeded by environmental *obstacles* (e.g., physical barriers, inaccessibility of services and amenities, social stress, and resource inadequacy), which can impact positive health, social participation and health equity. Indeed, individuals with disabilities need support from the social environment [34, 39] and accessibility in the physical neighbourhood environment [34, 39–42] to help them live in the community [34, 43, 44]. Moreover, the closure of nearby services has been shown to be worrisome [37], especially for women considering the prospect of not being able to drive or concerned about declining mobility [36].

As highlighted in a recent scoping study, mobility and social participation in older adults have been demonstrated to be positively associated with indicators of most age-friendly characteristics, i.e., with 1) proximity to resources and recreational facilities, 2) social support, 3) having a car or driver’s license, 4) public transportation, and 5) neighbourhood security, and negatively associated with 6) poor user-friendliness of the walking environment, and 7) neighbourhood insecurity [45]. Nevertheless, based on an analysis of cross-sectional data [46], environmental variables associated with social participation differed according to area. Indeed, only the social participation of older adults living in metropolitan areas was associated with transit and quality of the social network, whereas social participation in rural areas was correlated with the presence of children living in the neighbourhood and more years lived in the dwelling. Having a driver’s license and greater proximity or accessibility to resources were associated with social participation in all areas [46]. In addition to be associated with social participation, resources, transportation and social networks are among age-friendly issues.

#### How age-friendly communities foster positive health, social participation and health equity: What is needed for a better understanding?

Although age-friendly communities are believed to be a promising way to help older adults lead healthy and active lives and stay involved in their communities, it is essential to know which and how age-friendly key components of communities (i.e., policies, services and structures related to physical and social environments of communities) foster positive health, social participation and health equity [47]. Previous research focused mainly on comparing age-friendly approaches [48] or assessment [49], detecting factors that assist communities in or hinder them from becoming age-friendly [50], explaining the collaborative partnership conditions and factors that foster implementation effectiveness [51], as well as identifying priorities for actions [52]. This research provided valuable information on most common projects that, for example in Manitoba, were related to outdoor spaces, buildings, communications and activities (e.g., walking groups, contacting isolated older adults) [52]. However, projects vary across communities and change over time. Rural communities’ ability to become age-friendly was influenced by contextual factors such as size, location, demographic composition, ability to secure investments, and leadership [50]. Communities mostly assess their success by considering the level of community involvement, surveys, program attendance and number of classes taught, as well as incorporating the needs of older adults in organisations’ and agencies’ strategic planning [53]. Moreover, research has shown differences in rural versus urban communities’ trajectory and timeline for improving age-friendliness. On the one hand, some rural communities made quick progress with small projects but had more difficulty tackling larger projects than urban centres [52]. The presence of strong social ties and sense of place were found to be among their strengths, whereas poor infrastructure, widely dispersed population, large geographic distance, and aging as a result of out-migration were among their challenges [52]. Urban centres, on the other hand, may need more time in the early stages but, by building on existing infrastructures and processes, may be able to address larger projects more easily [52]. Finally, another cross-sectional study found that a superior rating of age-friendliness was associated with a higher percentage of residents aged 65 or older [54]. The communities that were identified as having the lowest age-friendliness were small communities located within a census metropolitan area and remote communities in the far north of Manitoba. Future studies are needed to determine whether age-friendly initiatives benefit all older adults and specifically their health [55].

To our knowledge, only one US [56] and three Canadian empirical studies, two in Manitoba [57, 58] and one in the province of Quebec [59], examined the influences of age-friendly communities on health-related outcomes. Using an exploratory factor analysis of items from a sample of 1376 urban older Americans, six factors were identified as being associated with demographic characteristics and self-rated health: 1) access to business and leisure, 2) social interaction, 3) access to health care, 4) neighbourhood problems, 5) social support, and 6) community engagement [56]. One Canadian study used photovoice with 30 participants in one urban and three rural age-friendly communities in Manitoba and found that to promote health and well-being and facilitate independent living, it is important to ensure that older adults have access to a broad range of community supports such as provision of services, counselling, congregate meals, volunteer drivers and a medical equipment-lending program [57]. For example, congregate meals are beneficial to those who live alone and have difficulty purchasing groceries, by providing not only needed nutrients but also opportunities for social interaction. Moreover, waiting lists for medical and long-term care are a key concern, and rural areas present unique challenges, with their transportation difficulties and greater proportion of older adults [57]. Since transportation links older adults not only with health services but also with community life including local businesses, services and opportunities for social participation, the absence of affordable and accessible transportation may create barriers to health services, contribute to social isolation and decrease health equity. Finally, in addition to transportation, multiple aspects of older adults’ lives, including housing, social environment, activities and volunteering, and community supports and health services are influenced by affordability [57]. The limitations of this study were the relatively small number and homogeneity of the participants, and its possible selection bias towards healthier and younger individuals living in only four age-friendly communities in Manitoba.

Another cross-sectional study involved a needs assessment process in 29 communities beginning age-friendly initiatives in rural Manitoba, where 593 younger and older adults completed a survey [58]. The survey focused on the presence or absence of *age-friendly key components* in seven domains targeted by the WHO [19] (physical environment, housing options, social environment, opportunities for participation, community supports and healthcare services, transportation options, communication and information), generating a total score and for each domain, as well as life satisfaction and self-rated health. The results indicated that a higher rating of the community’s age-friendliness was related to greater life satisfaction and self-rated health [58]. All of the seven age-friendly domains except housing were positively related to life satisfaction. For self-rated health, significant relationships emerged with physical and social environment, opportunities for participation, and transportation options [58]. Age-friendly domains’ relationship with life satisfaction and self-rated health were restricted primarily to older adults. None of the community characteristics [population size, % of residents 1) aged 65 or older, and 2) with less than high school education, and median income] were related to life satisfaction and self-rated health, nor was degree of rurality [58]. However, as the responses were made immediately after the age-friendly survey, life satisfaction and self-rated answers might have been influenced by that study. Moreover, limited information was available on participants’ sociodemographic characteristics, reducing the possibility to consider intrapersonal factors in the analyses. Participants were also from only one Canadian province (Manitoba), were not randomly selected and might not be representative of their communities. Finally, the communities involved were assessing their needs, i.e., in an early phase of the age-friendly community and prior to the implementation of initiatives.

Lastly, a realistic evaluation study is currently underway to identify how characteristics of four contrasted age-friendly communities in the province of Quebec (i.e. a city of over 100,000 inhabitants, a group of villages around a rural municipality, a rural municipality and a municipality in the suburbs of Montreal) influence social determinants of health and foster health equity [59]. Preliminary results show that these cases use a community development approach, with strong civic participation from older adults, intersectoral collaboration, governance, leadership, development of capacities of communities and individuals, and concrete actions involving older adults. Their supralocal consulting modes and intersectoral partnerships help to sustainably change access, supply or service organisation to improve the living conditions identified by older adults as priorities [59]. However, this study focuses on sectoral stakeholders and does not examine health issues of older adults living in these communities.

Despite the contribution of these studies, little is known about age-friendly key components of communities and how they foster positive health, social participation and health equity. Comparisons of communities according to health outcomes would also be important [58]. In addition, qualitative studies are needed for an in-depth exploration of how age-friendly initiatives impact older residents living in diverse settings [52]. A health equity lens is also needed to ensure that age-friendly communities not only improve health but also foster health equity. As age-friendly domains cannot be treated in isolation from intrapersonal factors (e.g., functional status) and other levels of influence (e.g., policy environment), the ecological premises underlying age-friendly communities also support the need for a holistic and interdisciplinary approach [60]. Two ecological models of health, one focusing on multiple aspects of the neighbourhood environment [61, 62] and one specific to age-friendly communities, have been developed recently to reduce communication difficulties and stimulate collaboration across disciplines (e.g., public health, rehabilitation and gerontology). Moreover, recent advances have been made in the conceptualisation (definition) and operationalisation (measurement) of positive health [5], social participation [63] and health equity [27]. Such efforts were required to enable optimal future research on the impact of age-friendly communities as a population intervention. It is thus timely, innovative and essential to conduct more research to *better understand which and how key components of age-friendly communities best foster positive health, social participation and health equity in aging Canadians*. Specifically, the research aims to:

1. Describe and compare age-friendly key components of communities across Canada
2. Identify key components best associated with positive health, social participation and health equity of aging adults
3. Explore how these key components foster positive health, social participation and health equity.

As part of the second objective, the correlates of positive health and social participation will be examined with a health equity lens. More specifically, the relationships between key components, positive health and social participation will be examined according to various individual-level characteristics (Table 1). Environmental characteristics will also be considered to quantify social and geographical inequalities (Table 1). The choice of these individual and environmental correlates is based on the literature, including one scoping review [45]. Age-friendly key components that enhanced both positive health and social participation of aging adults in these subgroups will be identified as they might foster health equity.

**Table 1:** Individual and environmental characteristics

Individual characteristics	Specifications	Environmental characteristics	Specifications
Age	Younger vs. older	% of residents	1) Aged 65 or older 2) With less than high school education 3) Living under the low-income threshold 4) Immigrant (i.e. born outside Canada)
Gender	Women vs. men		
Marital status	In a couple vs. alone		
Education	Higher vs. lower		
Income		Median income	Higher vs. lower
Socioeconomic status (SES)		Materially or socially deprived area	Less vs. more
Wealth		Population size	
Social status		Urban, rural or remote area	
Chronic condition	Less vs. more		
Pain			
Disability			
Stress			
Medications			
Falls			
Social support	More vs. less		
Driver’s license and means of transportation	Yes vs. no		

## Methods

### Design and participants

A mixed-method sequential explanatory design [64] (quantitative ➔ qualitative) will be used and embrace a participatory approach involving researchers from different disciplines, practitioners, and policymakers to mobilise and engage communities and age-friendly experts inside and outside the health sector across Canada. The *quantitative* part will be carried out in two steps. First, all Canadian communities, defined by the municipalities (*N* = 3555), will be surveyed to document and compare age-friendly key components across Canadian communities (*Objective 1*). A knowledgeable person identified by the mayor of the municipality will complete the online survey. Although many of them did not formally implement age-friendly initiatives, communities may still present some key components. Second, to identify the age-friendly key components best associated with positive health, social participation and health equity of aging adults (*Objective 2*), rigorous secondary analyses of available cross-sectional data (baseline) of the *Canadian Longitudinal Study on Aging* (CLSA) [65] will be conducted on all participants (*n*_respondents_ = 41,085) living in communities in which there are at least 30 CLSA respondents (*n*_municipalities_ = 185). This sample size is justified by multilevel analysis (see below). Specifically and regardless of whether they live inside or outside a community labeled as age-friendly, positive health, social participation and health equity from CLSA respondents will be related to age-friendly key components of their community. The CLSA is a large, national, long-term study representative of the Canadian population designed to examine health transitions and trajectories of adults as they age [65]. This Canada-wide study involves a total of 51,352 people between 45 and 85 years of age at baseline, living in private dwellings, and followed from 2013 for 20 years. A stratified random sampling strategy was used to recruit respondents based on age, gender, province, and rural or urban area [65]. All 51,352 participants were asked to provide a core set of baseline information on demographic and health measures. The information is collected on all participants via computer-assisted interviews completed in participants’ homes or via telephone interviews [65].

In the *qualitative* part, a multiple case study will be conducted in five Canadian communities performing best on positive health, social participation and health equity (*Objective 3*). A qualitative multiple case study design allows analysis and in-depth description of a social phenomenon [66]. In the current study, this design will make it possible to *consider contextual factors, i.e.* *, global, social, political, economic and cultural factors surrounding communities and the implementation of age-friendly initiatives, including variations and adaptations*. Participants (i.e., aging adults, caregivers and, if applicable, age-friendly community committee members), will be recruited using purposeful sampling to ensure heterogeneity of experience and based on individual and environmental characteristics identified in the quantitative part as being mainly associated with health equity challenges. The Research Ethics Committee of the *Centre integré universitaire de santé et de services sociaux de l’Estrie — Centre hospitalier universitaire de Sherbrooke* (*CIUSSS de l’Estrie-CHUS*), have approved the study.

### Quantitative part

#### Variables and instruments

The survey (*Obj. 1*) will include the age-friendly questionnaire targeting *key components* in seven domains: physical environment, housing options, social environment, opportunities for participation, community supports and healthcare services, transportation options, communication and information. This questionnaire includes 54 items answered yes/no/don’t know [54]. Sum of yes answers generates total and domain scores, higher scores representing greater age-friendliness. The total and seven domain scores present good internal consistency (Cronbach’s alpha = 0.63 to 0.86) [54]. In the same survey, community *implementation of age-friendly initiatives* will be categorised with the Pan-Canadian Age-Friendly Communities Milestones: 0) did not initiate age-friendly steps, 1) establish an advisory committee that includes the active engagement of older adults, 2) secure a local municipal council resolution to actively support, promote and work towards becoming age-friendly, 3) establish a robust and concrete plan of action that responds to the needs identified by older adults in the community, 4) demonstrate commitment to action by publicly posting the action plan, and 5) commit to measuring activities, reviewing action plan outcomes and reporting on them publicly.

The CLSA (*Obj. 2*) includes *positive health* measures such as life satisfaction, functional status and performance (Hand grip strength, Timed up-and-go; Chair rise, Standing balance, and 4 m walk), basic and instrumental activities of daily living (Older Americans Resources and Services), general health (general and mental health, and healthy aging), mobility pattern, health care utilisation (contacts with healthcare professionals) and physical activity (scale for the elderly) [65]. As it was not possible to carry them in telephone interview, functional status, performance and mobility pattern were available only among a subsample (*n* = 30,111) of the participants and will be analyzed in an explorative manner.

The *social participation* measure reports frequency of participating in eight activities: family or friends outside the household; church or religious; sports or physical; educational and cultural; service club or fraternal organization; neighbourhood, community or professional association; volunteer or charity work; and other recreational (e.g., hobbies, bingo and other games). Responses are converted into frequency of participation for each activity (“at least once a day” = 20; “at least once a week” = 6; “at least once a month” = 2; “at least once a year” = 1; and “never” = 0) [14, 46, 67, 68]. The sum of activities results in a total social participation score representing the number of community activities per month.

The CLSA also specifically considers *health equity* through SES measures including indicators of educational attainment, type of employment and hierarchical position in the workplace, income, and wealth. Wealth is measured by 12 questions about current income, investments, assets, and adaptation from CLSA and Survey of Health, Aging and Retirement in Europe (SHARE) to reflect the Canadian context. Perceived social inequality is measured using the MacArthur Scale of Subjective Social Status [69]. The scale asks on which rung of a 10-rung “social ladder” participants feel they stand. This scale has been validated and shown to predict health status and decline in health status over time in middle-aged adults [70, 71]. Other variables from the CLSA that will be considered are age, gender, education, marital status, income, SES and wealth, social status, chronic medical conditions, pain, disability, stress, medications, falls, social support, driver’s license and means of transportation. Moreover, the CLSA includes contextual data concerning social cohesion, neighbourhood, and environmental quality [65]. Indicators of social cohesion include voter turnout, recycling rates, volunteer organisations per capita, newspaper readership, stability, charitable donations, and feelings of safety. Indicators of neighbourhood quality will include general economic status, neighbourhood type, amenities for aging people, rental costs, vacancy rates, shopping facilities, crime rates, and vandalism [65]. Indicators for environmental quality will include green space, air and water quality, and climate. Finally, material and social deprivation indexes consider respectively the proportion of persons without a high school diploma, the proportion employed, and average personal income, and the proportion of persons living alone, separated, divorced or widowed, and single-parent families [72]. Residential population density will be defined as thousands of residents/km^2^.

#### Quantitative analysis

At the community level, municipality characteristics and key components will first be described using means with standard errors or percentages, according to the type of variable (continuous or categorical, respectively). Chi-square tests and ANOVAs followed by pairwise comparisons (with Bonferonni adjustment) will be performed to identify differences in key components (total score and score for each of the seven age-friendly domains; *Obj. 1*) with regard to 1) Statistics Canada classification as metropolitan (≥ 150,000 inhabitants), urban (< 150,000 and ≥10,000), or rural (< 10,000) [73], 2) percentage of residents aged 65 or older (above or below median), 3) residential density (above or below median), and 4) material and social deprivation (above or below median).

Next, regardless of whether they live inside or outside age-friendly communities, positive health, social participation and health equity from CLSA respondents (*n*_respondents_ = 41,085 from *n*_municipalities_ = 185) will be associated with age-friendly key components (from Obj. 1 and matched using the six-digit postal code of respondent’s residence) and other environmental characteristics described above. Hierarchical linear and nonlinear (multilevel) models will be created to verify the associations between key components and the dependent variables, i.e., positive health and social participation, as well as their interaction with health equity (*Obj. 2*). As mentioned, health equity will be examined by verifying the moderating effects of age, gender, education, marital status, income, SES, wealth, social status, chronic medical conditions, pain, disability, stress, medications, falls, social support, driver’s license and means of transportation on the associations between the key components and positive health or social participation. Since our data have a hierarchical structure (i.e. CLSA respondents nested within communities), a two-level model will be used. Level one will consider individual variables (positive health and social participation as well as their interaction with some health equity indicators) and level two will consider key components and other variables at the community level. Only interaction terms presenting a *P* value lower than 0.05 will be retained. Using random effects to control for other factors not known or not included, the model will first be adjusted with the independent variables, i.e., key components. Second, characteristics specifically related to the individuals will be added to the model and, finally, those related to the communities. Analyses will be conducted using SAS (v9.2), which accounts for the stratified random sampling strategy, and HLM (v7).

### Qualitative *part*

#### Data collection

For each of the five cases of Canadian communities performing best on positive health, social participation and health equity (*Obj. 3*), face-to-face focus groups preferably or, if unavailability for the groups, individual interviews lasting between 90 and 180 min will be conducted separately with aging adults, caregivers and age-friendly community committee members using a semi-structured interview guide. At least three focus groups per case will be conducted. Homogenous groups of about 8 aging adults, caregivers or age-friendly community committee members (*n total* = 120) will facilitate the discussion. Questions will include, for example: ‘Tell me about the age-friendly initiatives that promote health and social participation in your community. How are less advantaged aging adults reached by age-friendly initiatives? What help do you receive to carry out your activities, what resources do you use to conduct your affairs?’ Interviews will aim to identify how key components of age-friendly communities and their process produce positive health, social participation and health equity among aging adults. If needed, follow-up telephone interviews will clarify information. Interview guides will be reviewed by an external group of qualitative research experts and be adjusted during data collection [74]. All interviews will be audiotaped and transcribed. The research will also consider organisational documents, such as age-friendly communities’ action plans, and all participants will complete a sociodemographic questionnaire. Multiple information sources will be used to perform data triangulation and foster a holistic understanding of the situation.

#### Qualitative analysis

Participants and their context will first be described using means with standard errors, median and interquartile interval or frequencies with percentages, according to the type of variable (continuous or categorical, respectively) and number of participants. Focus groups and organisational documents will be analysed with thematic content analysis as described by Yin [66] using mixed extraction grids [75] to explore how the age-friendly key components and their process foster positive health, social participation and health equity among aging adults (*Obj. 2*). Themes emerging from the content of the interviews and organisational documents will only be organised afterwards and renamed according to the Human Development Model–Disability Creation Process, an anthropological model of human development and disability [16]. Interviews and organisational documents will be analysed individually (intracase) and syntheses will be written following analyses of each case. Intercase analyses will then be performed to identify similarities and differences. Cases will be discussed by the team and one-third will be co-coded. Memos including thoughts, questions and team discussions will be used. Analyses will be conducted using NVivo (v10).

## Discussion

First the potential outcomes of this project will be addressed. Finally, the strengths and limitations will be discussed.

### What outcomes are expected from this project?

Building on new and existing collaborations and generating evidence on key components and their setting, the results of this project will ultimately help communities, labeled as age-friendly or not, to refine policies, services and structures which best *foster positive health and promote social participation among all aging Canadians*. Age-friendly communities are among the most innovative population interventions with a high probability of enhancing health and health equity at a population level. These interventions might be designed according to asset-based approaches in which collective resources are made available for individuals and communities as a way to promote health status in a positive way [76]. Moreover, this project will mobilise researchers, practitioners and policy-makers working with aging populations inside and outside the health sector in a reflexive process. This reflection will ultimately lead to the consideration, development and application of age-friendly communities’ new evidence in health and other sectors, including in programs or services delivered in a primary health and social services context. In addition to further introducing the health promotion discourse in the health and social system, which is too often directed toward disease prevention rather than health promotion or clinical rather than population-based interventions, this mobilisation will contribute to helping aging Canadians enjoy good health and stay involved, to reducing inequalities and to increasing access to opportunities and conditions conducive to health for all.

### Strengths and limitations

This study will be carried out using *rigorous and complementary approaches of mixed-method designs*, including secondary analysis of one large cross-sectional database representative of the Canadian population and using *culturally appropriate, gender sensitive and ethically sound methods*. As the research team was not involved in developing and implementing the Canadian age-friendly initiatives, a neutral perspective will be adopted. Quantitative results will also be completed and extended by qualitative results [77], which will help to better understand how age-friendly communities promote positive health, social participation and health equity in aging Canadians. Enriched by a unique and close collaboration of knowledge-users from different fields in a variety of institutions, the results empirically support age-friendly cities. Through the synergy of an exceptional research team, the results will be exhaustively disseminated to knowledge-users and support equitable and ethical partnership with communities. A guide on collaboration between researchers and knowledge-users in health research is used to optimize the multidisciplinary partnership [78]. However, as in other secondary analyses studies, the current project will only consider variables of positive health, social participation and health equity available in the CLSA and Census. Analyses will also relate these variables to those from a survey collecting data from a more recent timeframe. The impact of these limitations should nonetheless be minimal since these surveys include required variables that will be combined with other environmental data and AFC are slowly modified in time. Although involving a significant number of communities and because of multilevel analysis, this proposal will focus on urban municipalities. Rural municipalities will be targeted in future studies by our team. Furthermore, the qualitative part of the study will involve only five cases, which will allow in-depth exploration, but not cover all initiatives.

## Abbreviations

CIHR: Canadian Institutes of Health Research
CLSA: Canadian Longitudinal Study on Aging
SES: Socioeconomic status
SHARE: Survey of Health, Aging and Retirement in Europe
US: United States
WHO: World Health Organisation
